# *Sargassum plagiophyllum* Ethanolic Extract Enhances Wound Healing by Modulating FAK/Src/Akt/p38 and Rac1 Signaling in Keratinocytes HaCaT Cells

**DOI:** 10.1155/adpp/7198281

**Published:** 2025-01-22

**Authors:** Furoida Moolsup, Wanida Sukketsiri, Wipawadee Sianglum, Jirawat Saetan, Nattakanwadee Khumpirapang, Supita Tanasawet

**Affiliations:** ^1^Division of Health and Applied Sciences, Faculty of Science, Prince of Songkla University, Songkhla 90110, Thailand; ^2^Laboratory Animal Service Center, Faculty of Science, Prince of Songkla University, Songkhla 90110, Thailand; ^3^Division of Biological Science, Faculty of Science, Prince of Songkla University, Songkhla 90110, Thailand; ^4^Department of Pharmaceutical Chemistry and Pharmacognosy, Faculty of Pharmaceutical Sciences, Naresuan University, Phitsanulok 65000, Thailand

**Keywords:** FAK, HaCaT cells, *Sargassum plagiophyllum*, Src, wound healing

## Abstract

Recently, seaweed extracts have been found to have potential in skin benefits. This study, therefore, aimed to explore phytochemical analysis, antimicrobial, antioxidant, and wound healing properties of brown seaweed *Sargassum plagiophyllym* ethanolic extract (SPEE) on human skin keratinocyte HaCaT cells and the possible mechanism involved. Our results indicated that SPEE contained flavonoid, phenolic, and carotenoid as the major active constituents. The HPLC chromatogram revealed C-phycocyanin and fucoidan presented in SPEE. SPEE demonstrated the antioxidant capability and significantly reduced wound space at 24 and 48 h in wound-healing assay. Treatment with SPEE (50 and 100 μg/mL) increased FAK and Src phosphorylation in western blotting. Moreover, SPEE also upregulated Akt and p38 MAPK phosphorylation but not ERK1/2. SPEE increased Rac1 protein expression. Interestingly, hyaluronan synthase (HAS1 and HAS2) as well as collagen type I and elastin were also significantly upregulated when compared with the control upon exposure to SPEE. In conclusion, our data suggested that SPEE promotes cutaneous wound healing by regulating FAK/Src-mediated Akt, p38 MAPK, and Rac1 signaling. These findings suggest the potential use of SPEE for skin wound treatment.

## 1. Introduction

Human skin composes of epidermis, dermis, and hypodermis, which possess the first line of defense against pathogens, physical, and chemical traumas [1]. Cutaneous wounds from trauma, burns, microbial infection, or diabetes leading to nonhealing wounds cause a significant burden to most patients and healthcare systems [2]. In response to skin injury, the process of dermal wound repair takes place. Wound healing comprises three stages: inflammation, proliferation, and remodeling. During the inflammatory phase, hemostasis and the recruitment of inflammatory cells occur. The proliferative phase relates to granulation tissue formation, subsequent re-epithelialization, and collagen deposition. Finally, the remodeling phase is associated with the maturation and reorganization of the collagen fibers. Epidermal cells are necessary to complete wound closure. Keratinocytes are the predominant cell type occupying all layers of the skin epidermis and actively participate in each phase of wound healing. They contribute to the immunological and inflammatory process, migrate, and proliferate in association with wound closure mechanism via re-epithelialization, and tissue maturation. Their dynamic functions are essential for the successful wound-healing process and the restoration of the skin's structural and functional integrity [1, 3, 4].

Marine algae-derived compounds have been demonstrated to be a beneficial source for treating skin-related diseases, including wounds, due to their good biocompatibility, nontoxicity, low cost, and availability [4–6]. Marine brown algae, *Sargassum* species, have been recognized to exert several health benefit potential such as antioxidant, anti-inflammation, anticancer, anticholesterol, and enhance colonic functions [6–10]. Biologically active constituents present in brown seaweeds are polysaccharides (alginates, laminarans, and fucoidans), pigments (such as carotenoid fucoxanthin), polyphenols (phlorotannins), and other compounds including vitamins, minerals, and lipids [6, 8, 11]. The application of natural biologically active compounds extracted from seaweed such as polysaccharides enhances the wound-healing process by preventing the microbial growths, accelerates wound closure time, stimulates the deposition of collagen type I fibers, and thereby promotes the re-epithelialization process both in vitro and in vivo studies [4–6, 12].

Recent studies have demonstrated that Sargassum species, including *Sargassum polycystum* and *Sargassum ilicifolium*, have the capacity to enhance cell proliferation and cell migration and exhibit wound-healing properties [13, 14]. However, previous studies have reported that the phytochemical composition and pharmacological activities of different seaweed species vary depending on geographical distribution and environmental factors [15–17]. This study thus aimed to evaluate the phytochemical, antimicrobial, antioxidant, and wound-healing potentials of *Sargassum plagiophyllym* ethanolic extract (SPEE), an abundant species found in Thailand, on human keratinocytes HaCaT cells. Importantly, we also elucidate the molecular mechanism behind wound-healing effects of SPEE on keratinocytes, which have not been previously reported.

## 2. Materials and Methods

### 2.1. Preparation of *Sargassum plagiophyllum* Extracts

*S. plagiophyllum* were obtained from Trang province, Thailand, and dried in an oven at 45°C prior to being ground into a powder. The dried *S. plagiophyllum* powder (1 kg) was macerated using 70% ethanol. The mixture was frequently stirred for 72 h at room temperature, after which it was filtered using Whatman No 1 filter paper. The solvent was then removed by evaporation. The yield of the *S. plagiophyllum* ethanolic extract (SPEE) was 10.72% (w/w) of the dried powder.

### 2.2. Evaluation of Total Phenolic, Flavonoid, and Carotenoid Contents

The total phenolic content was evaluated using the Folin–Ciocalteu assay. Twenty microliters of the extract were added into a 96-well plate, followed by the addition of 100 μL of 10% Folin–Ciocalteu reagent and 80 μL of 7% sodium carbonate. The mixture was incubated for 30 min and measured at a wavelength of 760 nm using a microplate reader (Synergy H, Bio-tex, VT, USA). Total phenolic contents of SPEE were expressed in terms of mg gallic acid equivalents per gram extract (GAE). The total flavonoid content of SPEE was examined using the aluminum chloride colorimetric method. Twenty microliters of SPEE was pipetted into a 96-well plate and incubated with the mixture of aluminum chloride and sodium acetate in dark for 15 min. The absorbance was read at a wavelength of 430 nm. The total flavonoid content was expressed in terms of milligram quercetin equivalents per gram extract (QE). In addition, the total carotenoid content was also determined by adding 100 μL of SPEE solution into a 96-well plate and measuring the absorbance at 450 nm using a microplate reader. The total carotenoids content was expressed in terms of milligram *β*-carotene equivalents per gram of extract (mg *β*-carotene/g extract).

### 2.3. Chromatographic System

SPEE was mixed with 0.1% trifluoroacetic acid (TFA), followed by filtered through a 0.45 μm membrane, and subsequently analyzed using high-performance liquid chromatography (HPLC) (Shimadzu LC-10ADvp, Kyoto, Japan) as previously described [13]. The HPLC was performed using the Shim-pack GISS C18 column at 4.6 × 150 mm and 5 μm of particle size. The determination of c-phycocyanin in SPEE was identified by using calibration curves for the standards of c-phycocyanin from Sigma-Aldrich (Sigma-Aldrich, USA). The signal detection was carried out using photodiode array detector (PDA) at 210 nm.

### 2.4. Determination of in Vitro Antibacterial Activity

The organisms used in this study were *S. aureus* ATCC29213, *E. coli* ATCC25922, *E. faecalis* ATCC29212, and *B. cereus* PSU414. Tryptic soy agar (TSA) and tryptic soy broth (TSB) were used for bacterial cultivation. Bacteria in glycerol stock was streaked on TSA agar plate and then incubated overnight at 37°C. Three to five bacterial colonies were inoculated into 5 mL TSB and incubated at 37°C for 4–6 h to reach the exponential growth phase. The isolates were then adjusted in Mueller Hinton broth (MHB) using a 0.5 McFarland standard for a bacterial total concentration of 1.5 × 10^8^ CFU/mL. Antibacterial activities of SPEE were investigated using the broth microdilution method, which was used to explore the minimum inhibitory concentration (MIC). It was performed using two-fold serial dilution in MHB in a 96-well plate as previously described [18]. The total volume in each well was 200 mL, and a 1% (v/v) DMSO was utilized as the negative control. The plates were then incubated for 16–18 h at 37°C. The bacterial growth was determined by the conversion of the blue dye resazurin to pink compound resorufin. The MIC is defined as the lowest antimicrobial agent that inhibits a color change of the resazurin. Ten mL of the concentration of MIC was inoculated into MHA plates to evaluate the minimum bactericidal concentration (MBC). MBC was verified as the lowest concentration of the compound that prevented the formation of bacterial colonies on the agar plate. Both MIC and MBC were calculated based on triplicate experiments.

### 2.5. Determination of In Vitro Antioxidant Activity

The 2,2-diphenyl-1-picrylhydrazyl (DPPH) method was employed to assess the free radical scavenging capabilities of SPEE. The DPPH solution (0.1 mM in methanol) was mixed with SPEE for 30 min in the dark and the absorbance was read at 517 nm using a Synergy microplate reader (Bio-tex, VT, USA). The 2,2-azinobis (3-ethylbenzothiazoline-6-sulfonic acid) (ABTS) assay was conducted following the previously described method and demonstrated as μmol Trolox/g extract [19]. The ABTS solution (2 mL) was mixed with 1 mL of SPEE and incubated for 10 min in the dark. The absorbance was measured at 734 nm using a microplate reader (Synergy H, Bio-tex, VT, USA). The ferric-reducing antioxidant power (FRAP) method was detected and represented as μM Fe^2+^/g sample (16). For this assay, 2.2 mL of FRAP reagent was incubated with 0.3 mL of SPEE for 15 min in the dark. The absorbance was then read at 593 nm using a Synergy microplate reader (Biotex, VT, USA). Metal chelating activity (MCA) was carried out using the previously described method and presented as μmol EDTA equivalents per gram sample (16). In brief, 1000 μL of SPEE was mixed with 50 μL of FeCl_2_ in a 96-well plate. Then, 200 μL of ferrozine was added for 10 min in the dark and measured at 562 nm using a Synergy microplate reader (Biotex, VT, USA).

### 2.6. Determination of the Wound-Healing Activity of SPEE in Keratinocyte HaCaT Cells

#### 2.6.1. Cell Viability Assay

Human keratinocyte HaCaT (CLS cell line service, Heidelberg, Germany) (1 × 10^4^ cells/well) were cultured into a 96-well plate with complete medium (DMEM medium containing 10% fetal bovine serum, 1% penicillin/streptomycin, and 1% L-glutamine) for overnight and then treated with SPEE at the concentrations of 1, 5, 10, 25, 50,100, 250, 500, and 1000 μg/mL for 24, 48, and 72 h. After incubation, 0.5 mg/mL of 3-(4,5-dimethylthiazol-2-yl)-2,5-diphenyl tetrazolium bromide (MTT; Thermo Fisher, USA) were added and leaved at 37°C for 2 h. The formazan product was dissolved with dimethyl sulfoxide (Lomachemie, India) and determined by a Synergy microplate reader (Biotex, VT, USA) at 570 nm.

#### 2.6.2. Scratch Wound-Healing Assay

The scratch wound-healing method was conducted to investigate the effect of SPEE on skin keratinocytes' migration. The cells (0.2 × 10^6^ cells/well) were seeded into a 6-well plate with complete DMEM medium until 100% confluence monolayer. Then, a sterile 200 μL pipette tip was used to create a wound space in the middle of the confluent cell monolayer and treated with SPEE at concentrations of 25, 50, and 100 μg/mL. The scratch width and wound area were captured at 0 h to establish a baseline and at subsequent time points of 24 and 48 h to quantify the percent wound closure compared with the initial scratched area using a phase-contrast inverted microscope, focusing on three points per line within the image field. The ImageJ software was used to measure the wound area at each time point and to calculate the percentage of the wound area relative to the initial wound area using the following formula: [average wound area at 24 or 48 h/average wound area at 0 h] *x* 100.

#### 2.6.3. Protein Expression by Western Blot Analysis

In order to examine cell division control protein 42 (Cdc42), Ras-related C3 botulinum toxin substrate 1 (Rac1), Ras homolog family member A (RhoA), p38 mitogen-activated protein kinases (p38), extracellular signaling-regulated kinases (ERK), protein kinase B (Akt), steroid receptor coactivator (Src), and focal adhesion kinase (FAK) protein expression, skin keratinocyte HaCaT (3 × 10^6^ cells/plate) were treated with SPEE at concentration of 25, 50, and 100 μg/mL for 24 h. Cells were then lysed with RIPA buffer containing protease inhibitor at 4°C for 30 min, centrifuged at 14,000 rpm for 20 min, and measured the concentrations of total protein by Bradford protein assay. Proteins (75 μg) were loaded to 10% sodium dodecyl sulfate (SDS)-polyacrylamide gel electrophoresis and transferred onto PDVF membranes. The membranes were first blocked using 5% skim milk and 3% bovine serum albumin (BSA). Following the blocking, the membranes were then incubated with primary antibody targeting Cdc42 (1:1000), Rac1 (1:1000), RhoA (1:1000), p38 (1:1000), p-p38 (1:1000), Akt (1:1000), pAkt (1:1000), ERK (1:1000), pERK (1:1000), Src (1:1000), pSrc (1:1000), FAK (1:1000), pFAK (1:1000), and ß-actin (1:1000) at 4°C overnight. Next, the secondary antibody conjugated with HRP was added and incubated for an additional 2 h. Finally, the specific protein bands were observed with chemiluminescence.

#### 2.6.4. mRNA Expression by Real-Time Reverse Transcription-Polymerase Chain Reaction (RT-PCR) Analysis

Total RNA was extracted by using TRIzol reagent (Thermo Fisher Scientific, USA) according to the manufacturer's protocol. cDNA was synthesized using SuperScript VILO™ cDNA synthesis kit (Thermo Fisher Scientific, USA). The mRNA expressions of hyaluronic acid synthase 1, hyaluronic acid synthase 2, hyaluronic acid synthase 3, collagen type 1 alpha 1 chain, and elastin were evaluated by real time RT-PCR using SensiFAST SYBR No-ROX kit (Bioline, UK). All data were analyzed by normalized gene expression using the 2^−ΔΔCT^ method. Primer sequences were shown in our previous report [13].

### 2.7. Statistical Analysis

The experiments were performed in triplicates and the data were expressed in as the mean ± standard error of the mean (S.E.M). Statistical analysis was performed using one-way analysis of variance (ANOVA) followed by LSD post hoc test. A *p* value < 0.05 was considered statistically significant.

## 3. Results

### 3.1. Phytochemical Analysis

**Figure 1:**
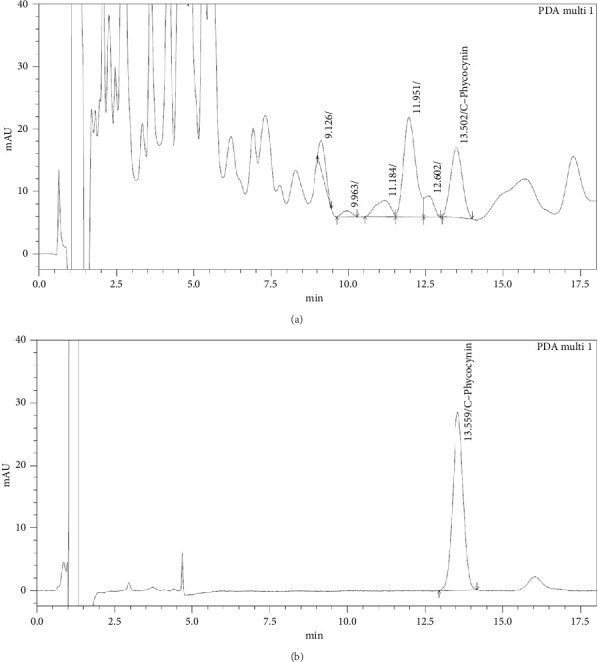
HPLC analysis of SPEE with the peak of c-phycocyanin (a) and c-phycocyanin standard (b).

The total phenolic, flavonoid, and carotenoid content present in SPEE were evaluated to determine the phytochemical constituents, as demonstrated in Table 1. The analysis found that the major constituents were flavonoid, phenolic, and carotenoid, with values of 102.13 ± 3.11 mg/g QE, 29.06 ± 0.04 mg/g GAE, and 1.45 ± 0.01 mg/g *β*-carotene, respectively. The HPLC chromatogram revealed a peak at a retention time of approximately 13.56 ± 0.15 min for C-phycocyanin (Figure 1). The contents of C-phycocyanin in SPEE were found to be 2.22 ± 0.02% w/w.

### 3.2. Determination of In Vitro Antibacterial Activity

The antimicrobial properties of SPEE were determined against four bacterial strains of *Staphylococcus aureus*, *Escherichia coli, Enterococcus faecalis*, and *Bacillus cereus*. The results showed that SPEE exhibited weak antibacterial activity against all four pathogens, with MIC and MBC above 2048 μg/mL, as shown in Table 2.

### 3.3. Determination of In Vitro Antioxidant Activity

The antioxidant activity of SPEE was evaluated using various in vitro assays, including the ABTS radical quenching assay, DPPH radical scavenging assay, ferric ion reducing antioxidant power (FRAP) assay, and metal chelating ability assay. As shown in Table 3, SPEE exhibited antioxidant capacity by quenching the ABTS radical and scavenging the DPPH radical at values of 102.23 ± 2.26 μmol Trolox/g extract and 67.24 ± 0.87 μmol Trolox/g extract, respectively. While the reducing power associated with the reduction of ferric ion to ferrous ion by the FRAP assay was 98.87 ± 3.78 μmol Fe^2+^/g sample, and the metal chelating ability was 46.87 ± 2.73 μmol EDTA/g sample, respectively.

### 3.4. Wound-Healing Activity of SPEE

#### 3.4.1. Cell Viability

**Figure 2:**
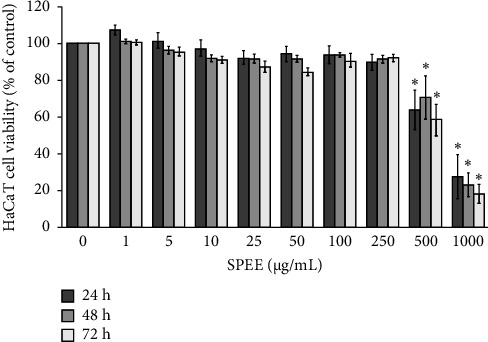
Percentage of cell viability of keratinocytes HaCaT cells exposed to various concentrations of SPEE (0–1000 μg/mL) at 24, 48, and 72 h. The cell viability was examined by MTT assay. The data are expressed as mean of each concentration ± SEM (*N* = 4). ⁣^∗^*p* < 0.05 as compared with the control group.

Treatment with SPEE at concentrations of 0–250 μg/mL for 24, 48, and 72 h did not cause any cytotoxic effect against keratinocyte HaCaT cells. However, higher concentrations (500 and 1000 μg/mL) showed a significant cytotoxic effect on the cells at 24, 48, and 72 h of treatment (Figures 2(a) and 2(b)). Therefore, the concentration range between 0 and 100 μg/mL was applied in further experiments because it proved to be nontoxic to the cells.

#### 3.4.2. Migration

**Figure 3:**
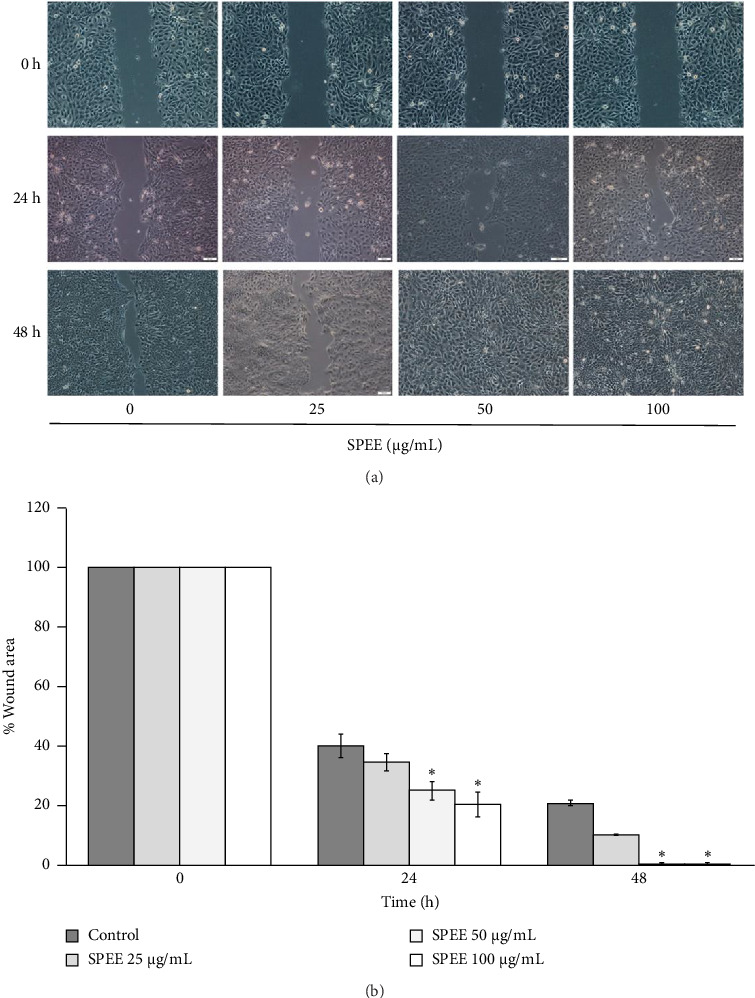
HaCaT keratinocytes scratch wound closure after treatment with SPEE. Microscopic images of wound gaps in HaCaT cells after exposure to different concentrations of SPEE (0, 25, 50, and 100 μg/mL) at 24 and 48 h (a). The percentage of wound area after exposure to different concentrations of SPEE (0, 25, 50, and 100 μg/mL) at 24 and 48 h (b). The data are expressed as the mean of each concentration ± SEM (*N* = 4). ⁣^∗^*p* < 0.05 as compared with the control group.

We next investigated whether SPEE promotes in vitro wound-healing potential by using a scratch wound model. The results revealed that exposure of SPEE to HaCaT cells at concentrations of 50 and 100 μg/mL significantly decreased the wound spaces to 25.10 ± 2.99 and 20.18 ± 4.09% at 24 h and complete the wound closure at 48 h, respectively, when compared with the untreated control as illustrated in Figures 3(a) and 3(b). However, no significant differences were observed at a concentration of 25 μg/mL after 24 and 48 h of treatment in HaCaT cells (Figure 3).

#### 3.4.3. SPEE Induced Cell Migration Through Activation of FAK/Src

**Figure 4:**
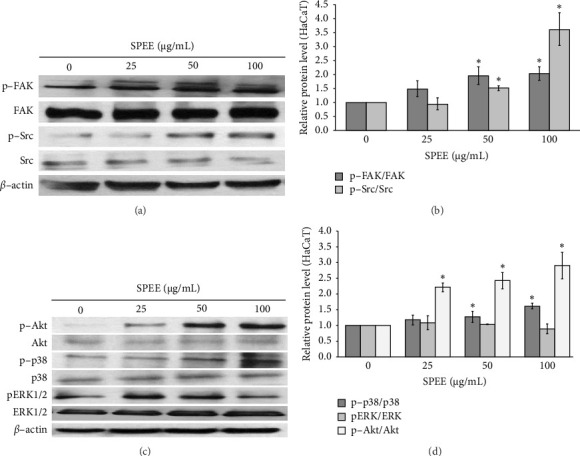
Effects of SPEE on the protein expression levels of p-FAK/FAK, p-Src/Src, p-Akt/Akt, p-p38/p38, and pERK/ERK in HaCaT cells. Cells were incubated with 25, 50, and 100 μg/mL SPEE for 24 h and further analyzed by western blotting. The data are expressed as the mean of each concentration ± SEM (*N* = 4). ⁣^∗^*p* < 0.05 as compared with the control group.

We examined whether SPEE affects the phosphorylation of FAK and Src, which play a crucial role in regulating keratinocyte migration and wound healing by western blotting. In HaCaT cells, treatment with SPEE (50 and 100 μg/mL) significantly increased the protein expression of p-FAK and p-Src as shown in Figures 4(a) and 4(b). However, at a dosage of 25 μg/mL, no significant difference was observed in HaCaT cells (Figure 4). These findings suggest that 50 and 100 μg/mL of SPEE can mediate the migratory activity of HaCaT keratinocytes via the activation of the FAK/Src signaling pathway.

#### 3.4.4. SPEE-Activated FAK/Src Induces the Phosphorylation of Akt and p38 MAPK

We further attempt to determine the possible cause of cell migration upon the exposure of SPEE in keratinocytes cells in the wound-healing process. Western blot analysis was used to explore the phosphorylation status of ERK1/2, Akt, and p38 after incubation with SPEE (0–100 μg/mL) for 1 h. Figures 4(c) and 4(d) show that SPEE significantly induced the phosphorylation of Akt in a dose-dependent manner, with the activation peak reaching 3 folds at 100 μg/mL. In addition, SPEE treatment at concentrations of 50 and 100 μg/mL markedly upregulated the phosphorylated p38 protein expression, while there was no significant effect on ERK ½ phosphorylation, as shown in Figure 4. These findings suggest that 50 and 100 μg/mL of SPEE can promote the migratory activity of keratinocytes by activating the FAK/Src signaling and also by inducing the phosphorylation of Akt and p38.

#### 3.4.5. SPEE-Activated Rac1 Expression

**Figure 5:**
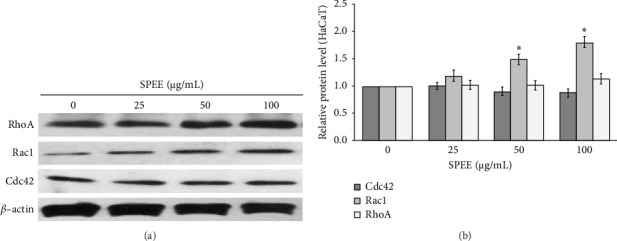
Effects of SPEE on the protein expression levels of Rho GTPase in HaCaT cells. Representative western blotting of RhoA, Rac1, and Cdc42 in response to 25, 50, and 100 μg/mL SPEE treatment (a) and immunoblots quantification of RhoA, Rac1, and Cdc42, respectively (b). All bands were quantified and normalized by *β*-actin. The data are expressed as the mean of each concentration ± SEM (*N* = 4). ⁣^∗^*p* < 0.05 as compared with the control group.

Next, we explored the involvement of downstream effectors three small Rho GTPases members, including Cdc42, Rac1, and Rho A, which have been linked to cytoskeletal organization during tissue repair. Western blot analysis showed that keratinocyte HaCaT cells treated with 50 and 100 μg/mL of SPEE exhibited a significant increase in only Rac1 protein expression, while there were no changes in Cdc42 and Rho A expression (Figures 5(a) and 5(b)).

#### 3.4.6. SPEE Activated Hyaluronan Synthase (HAS), Collagen Type I, and Elastin Expression

**Figure 6:**
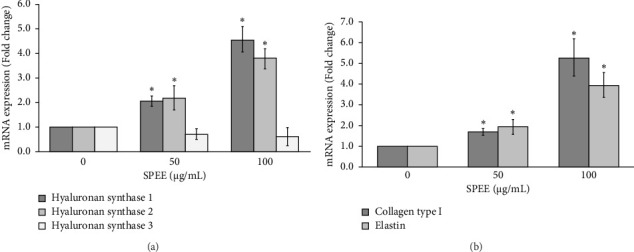
Effects of SPEE on mRNA expression of hyaluronan synthase (HAS1-3), collagen type I, and elastin. Cells were incubated with 50 and 100 μg/mL SPEE for 24 h, and total RNA was extracted and analyzed by real-time RT-PCR. The data indicate the fold change in expression relative to the control. The data are expressed as mean of each concentration ± SEM (*N* = 4). ⁣^∗^*p* < 0.05 as compared with the control group.

The extracellular matrix (ECM) plays a crucial role during the remodeling phase of wound healing. In this study, the effects of SPEE on ECM mRNA expression in HaCaT cells using real-time RT-PCR were evaluated. Our result revealed that HAS1 and HAS2 isoform were significantly upregulated after the administration of 50 and 100 μg/mL SPEE; however, HAS3 mRNA expression remained unchanged compared to the control group (Figure 6(a)). Meanwhile, SPEE at concentrations of 50 and 100 μg/mL significantly upregulated the level of collagen type I (1.70 ± 0.16 and 5.28 ± 0.91) and elastin (1.96 ± 0.37 and 3.98 ± 0.59) expression (Figure 6(b)).

## 4. Discussion

In recent years, there has been a growing interest in the development of wound dressing or drugs derived from seaweed for wound healing therapy. Brown seaweed has been demonstrated to possess wound-healing ability and anti-inflammation due to its rich source of bioactive compounds, making it a promising natural wound care [13, 14]. In the present study, we examined the phytochemical, antimicrobial, antioxidant, and wound-healing effects of brown algae, SPEE, on human keratinocyte HaCaT cells. In addition, we aimed to explore the underlying mechanisms of the wound-healing effects upon the treatment with SPEE. Based on our present findings, SPEE was found to contain the highest amount of flavonoids, which was consistent with previous research on *Sargassum polycystum* [13]. However, the highest phenolic content was observed in *Sargassum angustifolium* [20]. The differences in the phytochemical composition of the extracts might be attributed to the different extraction methods and the geographical region where the seaweed was sourced [15–17]. After further investigation using HPLC chromatography, we identified the presence of c-phycocyanin and fucoidan in SPEE. C-phycocyanin is a protein-bound pigment found in some microalgae, for example, spirulina. It has been reported to exert anti-inflammatory activities by inhibiting nitric oxide (NO) and prostaglandin E2 (PGE2) via the downregulation of inducible nitric oxide synthase (iNOS) and cyclooxygenase-2 (COX-2) expression. C-phycocyanin also diminishes the expression of tumor necrosis factor-alpha (TNF-*α*) and decreases neutrophils infiltration to the inflammatory sites [21]. Fucoidan, a sulfated polysaccharide commonly present in brown seaweed, has been found to enhance angiogenesis by the activation of Akt/Nrf2/HIF-1*α* signaling, thus facilitating the process of wound repair [22]. SPEE demonstrated weak antimicrobial activity against *S. aureus*, *E. coli, E. faecalis*, and *B. cereus*, which was in contrast with other studies. This contrary might possibly be attributed to solvent extraction, chemical composition, habitat, environmental factors, or place and season of seaweed collection [23]. Previous studies reported that antimicrobial activity is maximum in spring [24]. On the other hand, some studies have reported that the methanol extract showed strong bacterial inhibition percentage due to its high polarity [25]. Oxidative stress is known to disrupt or delay wound-healing processes, particularly by prolonging inflammation, inhibiting cell migration, and re-epithelialization [26]. Interestingly, our study indicated that SPEE exhibited antioxidant activity, as measured by various assays (DPPH, ABTS, FRAP, and metal chelating activity). This antioxidant activity likely contributes to the ability of SPEE to reduce oxidative stress, neutralize free radicals, and chelate metal ions. Studies have shown the positive effects of flavonoids, known nonenzymatic antioxidants, on wound healing due to their high hydroxylation. Thus, the presence of flavonoid in SPEE might be crucial in promoting wound healing [27].

Keratinocytes, as the major cellular component of the epidermal layer of the skin, play a significant role in the wound-healing process, being involved in the initiation, maintenance, and complete wound healing. The involvement of keratinocytes in wound healing encompasses various cellular and molecular events, including proliferation, migration, and epithelialization. In our present study, the wound-healing potential of SPEE has been studied on keratinocytes HaCaT cell line and has been found to enhance cell migration and wound closure without causing any cytotoxicity on keratinocytes. During the process of wound repair, it is well established that the activation of FAK/Src play a crucial role in regulating keratinocyte migration and proliferation to cover the wound space and reconstitute epidermal barrier [28, 29]. Our findings demonstrated that SPEE could increase the phosphorylation of FAK and Src in a concentration-dependent manner. FAK is a nonreceptor tyrosine kinase combining with both integrin *α*3*β*1 and various signaling pathways. The phosphorylation of activated FAK (Y397), which is a binding site for the SH2 (Src-homology2) domain of Src family kinases, thereby recruits Src and generates the activated FAK/Src complex to induce downstream signal transduction, such as p38 MAPK, Akt, and ERK [29, 30].

Previous study demonstrated that interleukin-6 (IL-6) activated p38 MAPK and its downstream CREB as well as Akt phosphorylation in nondiabetic wounds. The use of SB203580, a selective p38 MAPK inhibitor, was found to inhibit IL-6 promoted cell migration [31]. Immediately after corneal epithelial wound, p-p38 was upregulated near the wound edge, while p-ERK1/2 was undetectable, which is similar with this finding [32]. Treatment with SPEE (50 and 100 μg/mL) induced Akt and p38 MAPK phosphorylation but not ERK. These results suggested that SPEE induced FAK and Src phosphorylation, thereby promoting downstream Akt, p38 MAPK signaling, and keratinocyte migration. These findings are correlated with the observed phenotypic changes in the scratch wound-healing assay.

During the re-epithelialization process, the activation of FAK/Src has been found to regulate Rac 1, a member of the Rho family of small GTPases, to protrude lamellipodia at the leading edge and causes a forward movement of cells. In addition to Rac1, Rho A is known to activate the actin–myosin filaments to form stress fibers, while Cdc 42 is essential in maintaining cellular polarity [33, 34]. Our study demonstrated that SPEE stimulated only Rac 1 protein expression, while no differences were observed in Cdc42 and RhoA expression. These findings suggested the role of FAK/Src in coordinating Rac1, contributed to keratinocyte migration during wound-healing processes.

The skin ECM is composed of fibers and ground substances, including hyaluronan, that are important for structural scaffolding, elasticity, and homeostasis. Hyaluronan, a nonsulfated glycosaminoglycan, is synthesized by a family of transmembrane hyaluronan synthases (HAS1-HAS3). Collagen type I is stimulated by TGF-*β*1 and undergoes structural modification to restore the tensile strength of the healing wound in the remodeling phase. On the other hand, elastin plays a key role in the elastic recoil of ECM and activates fibroblasts to deposit new matrix in acute and chronic wounds [35, 36]. Exposure to SPEE was found to increase the mRNA expression of ECM-related molecules, including hyaluronan synthases (HAS1 and HAS2), collagen type I, and elastin. However, the mRNA expression of HAS 3 did not show any significant differences compared to the untreated control. Previous studies revealed that HAS1 was upregulated during keratinocyte differentiation under normal condition, while HAS3 synthesized low molecular weight polymers, which are mainly expressed in inflammatory or pathological condition [37, 38].

## 5. Conclusion

This study provides mechanistic insights into the wound-healing ability of SPEE, which is associated with its activation of the FAK/Src-mediated signaling cascade, including the Akt, p38 MAPK, and Rac1 pathways, as well as its antioxidant activity. However, a limitation of this study is that it is based on in vitro experiments; therefore, further in vivo research and clinical trials are essential to confirm the effectiveness of SPEE in wound healing in both normal and pathologic wounds.

## Figures and Tables

**Table 1:** The total phenolic, flavonoid, and carotenoid contents in SPEE.

Extract	Total phenolic content (mg gallic acid equivalent/g extract)	Total flavonoid content (mg quercetin equivalent/g extract)	Total carotenoid content (mg *β*-carotene/g extract)
SPEE	29.06 ± 0.04	102.13 ± 3.11	1.45 ± 0.01

*Note:* Values are mean ± SEM (*n* = 3).

**Table 2:** Antibacterial activity of SPEE.

Bacterial strains	MIC	MBC
*S. aureus* ATCC25923	> 2048 μg/mL	> 2048 μg/mL
*E. coli* ATCC25923	> 2048 μg/mL	> 2048 μg/mL
*E. faecalis* ATCC29212	> 2048 μg/mL	> 2048 μg/mL
*B. cereus* PSU 414	2048 μg/mL	> 2048 μg/mL

**Table 3:** Antioxidant activity of SPEE.

Extract	ABTS radical scavenging activity (μmol trolox/g extract)	DPPH radical scavenging activity (μmol trolox/g extract)	FRAP (μmol Fe^2+^/g sample)	Metal chelating activity (μmol EDTA/g sample)
SPEE	102.23 ± 2.26	67.24 ± 0.87	98.87 ± 3.78	46.87 ± 2.73

*Note:* Values are mean ± SEM (*n* = 3).

## Data Availability

The data that support the findings of this study are available on request from the corresponding author.
